# Multi-level manifestations of sexual stigma among men with same-gender sexual experience in Ghana

**DOI:** 10.1186/s12889-023-15087-y

**Published:** 2023-01-24

**Authors:** Khalida Saalim, Prince Amu-Adu, Richard Panix Amoh-Otu, Ransford Akrong, Gamji Rabiu Abu-Ba’are, Melissa A. Stockton, Richard Vormawor, Kwasi Torpey, Laura Nyblade, LaRon E. Nelson

**Affiliations:** 1 RTI International, Research Triangle Park, NC USA; 2 Priorities on Rights & Sexual Health, Accra, Ghana; 3 Youth Alliance for Health & Rights, Kumasi, Ghana; 4 Educational Assessment and Research Center, Accra, Ghana; 5 University of Rochester, Rochester, NY USA; 6 Department of Psychiatry, Columbia University Vagelos College of Physicians and Surgeons, New York, NY USA; 7 New York State Psychiatric Institute, New York, NY USA; 8 University of Ghana, Legon, Ghana; 9 Yale School of Nursing, New Haven, CT USA; 10 Yale Institute for Global Health, New Haven, CT USA; 11 Yale Center for Interdisciplinary Research on AIDS, New Haven, CT USA; 12 Yale School of Public Health, New Haven, CT USA

**Keywords:** Men who have sex with men, MSM, Anticipated stigma, Perceived stigma, Enacted stigma, Internalized stigma, Ghana, West Africa, Sexual stigma, Same-gender sexuality

## Abstract

Sexual stigma and discrimination toward men who have same-gender sexual experiences are present across the globe. In Ghana, same-gender sexual desires and relationships are stigmatized, and the stigma is sanctioned through both social and legal processes. Such stigma negatively influences health and other material and social aspects of daily life for men who have sex with men (MSM). However, there is evidence that stigma at the interpersonal level can intersect with stigma that may be operating simultaneously at other levels. Few studies provide a comprehensive qualitative assessment of the multi-level sexual stigma derived from the direct narratives of men with same-gender sexual experience. To help fill this gap on sexual stigma, we qualitatively investigated [1] what was the range of sexual stigma manifestations, and [2] how sexual stigma manifestations were distributed across socioecological levels in a sample of Ghanaian MSM. From March to September 2020, we conducted eight focus group discussions (FGDs) with MSM about their experiences with stigma from Accra and Kumasi, Ghana. Data from the FGDs were subjected to qualitative content analysis. We identified a range of eight manifestations of sexual stigma: (1) gossiping and outing; (2) verbal abuse and intrusive questioning; (3) non-verbal judgmental gestures; (4) societal, cultural, and religious blaming and shaming; (5) physical abuse; (6) poor-quality services; (7) living in constant fear and stigma avoidance; and (8) internal ambivalence and guilt about sexual behavior. Sexual stigma manifestations were unevenly distributed across socioecological levels. Our findings are consistent with those of existing literature documenting that, across Africa, and particularly in Ghana, national laws and religious institutions continue to drive stigma against MSM. Fundamental anti-homosexual sentiments along with beliefs associating homosexuality with foreign cultures and immorality drive the stigmatization of MSM. Stigma experienced at all socioecological levels has been shown to impact both the mental and sexual health of MSM. Deeper analysis is needed to understand more of the lived stigma experiences of MSM to develop appropriate stigma-reduction interventions. Additionally, more community-level stigma research and interventions are needed that focus on the role of family and peers in stigma toward MSM in Ghana.

## Background

Sexual stigma and discrimination toward men who have same-gender sexual experiences are present across the globe [1–4]. In Ghana, it is estimated that approximately 55,000 men participate in same-gender sexual activities [5]. In Ghana, same-gender sexual desires, relationships, and practices are stigmatized, and the stigma is sanctioned both through social and legal processes [6]. A recent national population-based survey in Ghana found that an overwhelming majority (90%) of Ghanaians believed that same-gender sexualities were inconsistent with Ghanaian cultural norms, undermined the basic social structure of Ghanaian society, and were therefore unacceptable [7]. It is well established that stigma is a social determinant that negatively influences health and other material and social aspects of daily life in the populations subjected to it [8–11]. In Ghana, the nearly ubiquitous presence of stigma and discrimination toward men with same-gender sexual experiences has the potential to limit the opportunities for these men to achieve and maintain equity in health, well-being, socio-emotional development, and economic progress [12, 13].

Sexual stigma is the social status devaluation of sexualities (e.g., desires, behaviors, identities, relationships, and communities) that are perceived and treated as abnormal because they are not heterosexual [14–16]. Sexual stigma in Ghana, as in many other countries around the world, is influenced by social policies and practices that preferentially treat heterosexuality as the “natural” or “normal” sexuality [12]. Sexual stigma is activated to socially relegate and regulate same-gender sexual practices among men because of those practices’ perceived incongruence with legal, cultural, and religious norms of masculinity [17, 18]. For example, a Ghanaian legal statute criminalizes “unnatural” sexual activities, which serves as a de facto legal premise that justifies stigma against same-gender sex because such sex is characterized as unnatural and unlawful, and thus socially dishonorable [6, 19]. Data on sexual stigma toward men who have sex with men (MSM) in Ghana align with data from research in other African contexts that suggest sexual stigma is grounded in religious and cultural perceptions around same-gender sexual behavior [20]. Moreover, in some African contexts, stigma towards MSM can be more pronounced among men who have gender non-conforming behaviors, or rather, act more feminine [21].Sexual stigma can be harmful to both physical and mental health, as evidenced by research conducted across the African continent that shows that stigma is in part responsible for lower rates of testing for HIV and higher levels of depression for MSM [2, 22]. Sexual stigma also influences health care seeking behavior by influencing MSM to either delay or forgo needed health care services [6]. In a seminal study, MSM in Ghana reported they felt they could not share their sexual identity while seeking care at a health care facility (HCF) and, instead, preferred not to seek care to avoid the discomfort [6]. Although the avoidance of HCFs is a protective behavioral strategy used by MSM to avoid exposure to sexual stigma [6], it can have indirect residual impacts on their health through either the lack of detection or delayed detection and treatment of chronic or communicable disease [6, 23].

Research describing sexual stigma among men with same-gender sexual experiences in Ghana is limited and has focused primarily on occurrences at the interpersonal level [6, 24–26]. However; there is evidence that stigma at the interpersonal level can intersect with stigma that may be operating simultaneously at structural and organizational levels [27]. The interactions among these multiple levels of sexual stigma are important to identify and understand because they may undermine the development of positive self-concept and maintenance of psychological well-being among the men [28–30]. Moreover, understanding multi-level sexual stigma within the Ghanaian sociocultural context can provide evidence to inform the development, testing, and implementation of socioculturally responsive stigma-reduction intervention strategies. Nonetheless, few studies provide a comprehensive qualitative assessment of the multi-level sexual stigma derived from the direct narratives of Ghanaian men with same-gender sexual experience. To help fill this gap in the state of the science on sexual stigma, our aim was to qualitatively investigate the following research questions: (1) what was the range of sexual stigma manifestations, and (2) how sexual stigma manifestations were distributed across structural, interpersonal, and intrapersonal ecological levels in a sample of Ghanaian MSM.

### Specifying terminology and positionality

A variety of terms have been used to describe men who have a range of same-gender experiences, including same-gender sex, desires, relationships, and identities [31]. These terms, such as ‘gay’, ‘bisexual’, ‘queer’, and ‘same-gender loving’ have specific sociopolitical and cultural meanings for the groups that identify with them [32, 33]. Since the onset of the HIV pandemic, the term men who have sex with men (MSM) has been broadly applied to men across the range of same-gender sexual experiences. MSM is an epidemiological term used in public health surveillance in the taxonomy of HIV exposure categories, such as perinatal or injection drug use exposures [34]. The term MSM does not refer to sexuality and can include, for example, a heterosexual man who was exposed to HIV through a non-consensual sexual encounter with another man. Over time, the use of “MSM” has crept beyond its original intention and is now popularly used as a convenient blunt short-hand reference to “gay” or “bisexual” [35]. The continued consolidation of men with diverse same-gender sexual experiences into a single yet epidemiologically precise term such as ‘MSM” erases important lived social realities of the men who are taxonomized into one group [35–37]. As a multicultural (Ghanaian, Korean, American) team led by Americans who are conducting U.S. Government-sponsored research in Ghana, we also acknowledge the settler colonial history of taxonomizing humans (in Africa, Asia, the Americas, and elsewhere) into groups in order to render them and their sociocultural practices more interpretable and manageable to individuals viewing and administering them through a Western intellectual lens [38, 39]. Except when making references to specific sexual identity groups, we use ‘MSM’ in this paper to refer to the men in the qualitative sample who were recruited in a larger HIV-prevention trial based solely on their self-reported potential HIV risk exposure category classification, not on their sexualities [40]. We offer this clarification here as an act of resistance to common practices that uncritically reduce men into behaviors as “MSM”, a dehumanizing practice that is entrenched in HIV-prevention literature [41] and one which is admittedly observable in our previous publications [24, 42–44].

## Methods

### Design

We used qualitative description as the overall design for the study. Qualitative description is a low-inference method that is well-suited for providing a summary of perspectives that remain anchored in the everyday language of the informants [45–47]. Our team has used qualitative description in previous studies [48], including research with MSM in Ghana [49, 50]. We also incorporated a community-based participatory approach by co-designing this qualitative sub-study with Ghanaian MSM community members and Ghanaian research assistants, including collaboratively identifying the research questions, analyzing the data, and writing this manuscript. Data were generated using focus group discussions (FGDs) that were conducted during the formative phase of a larger cluster randomized-controlled trial of a multi-level intersectional stigma-reduction intervention [40]. All study personnel were trained on the study protocol and ethics of research with human participants[51].

### Setting, sample and recruitment

The study was conducted in Accra, which is Ghana’s administrative capital, commercial center, and largest city, and in Kumasi, Ghana’s second largest city. Both areas are characterized by ethnic and religious diversity, including immigration from other regions of Ghana and neighboring countries. A convenience sample (*n* = 61) was recruited using the snowball technique. Initial participants were recruited by trained staff who had shared lived experiences as well as who had previous work experience conducting lesbian, gay, bisexual, and transgender (LGBT) community outreach. Individuals initially contacted were encouraged to invite others from their networks who might be interested. We obtained written informed consent from all participants. Inclusion criteria included being at least 18 years old, self-identifying as a cisgender man at time of enrollment, and self-reporting having had a sexual experience with another man (cis and transgender inclusive) within six months prior to enrollment. All participants received 100 cedis each for their participation in the study.

### Data collection

We conducted eight FGDs from March 2020 to September 2020, with six to eight participants in each group. FGDs were co-facilitated by pairs of trained research assistants. The FGDs were primarily conducted in English; however, each co-facilitator pair was multi-lingual in order to allow the participants to spontaneously communicate in the main indigenous Ghanaian languages (i.e., Twi, Ga). The FGDs were conducted in secure conference rooms of local LGBT community-centered non-governmental organizations. The discussions lasted between 90 and 120 min and were recorded as digital audio for transcription. All participants were asked to use pseudonyms.

Semi-structured guides were used to standardize the content and sequence of questions across FGDs[52, 53]. FGDs explored three main topics: (1) community norms, attitudes, and experience of life in their community; (2) experiences seeking or obtaining health care; (3) experiences of stigma in health care facilities. Sample questions and their associated prompts within each topic were used to encourage in-depth exploration, presented in Table 1. Although questions regarding family and peers were not formally included in the discussion guides, mentions of experiences of stigma among these social ecological groups also arose in the conversation and were probed and explored as they emerged [53, 54].

**Table 1:** Discussion topics, questions, and sample prompts from focus groups

Topic Domain	Question	Sample Prompts
Community norms, attitudes, and experiences of life	How would you describe the community where you live?	• What has it been like for you as a man who has sex with men? • What have been your most meaningful experiences in this community?
	Tell us about any times that you felt guilty or ashamed for having sex with other men?	• Where do you think these negative feelings come from? • How do these feeling affect your life? • What do you do to feel better about yourself and your sexuality?
Experiences seeking/obtaining healthcare	Who do you go see when you have a sexual health issue?	• What do you like about the provider that you visit? • What don’t you like about the experience? • Do you feel like you have a say in what happens to you there?
	Do you think that your health care provider cares about what happens to you?	• Why do you think so? • Why don’t you think so?
	When you leave the health care office, how capable do you feel to follow the provider’s instructions?	• Do you want to do what they advise? Why? Why not? • What keeps you from following through with the treatment plan?
Experiences of stigma in health care facilities	When you go for a health visit, what happens once the staff find out that you are MSM?	• Do you experience welcoming and positive reactions? • Have you experienced negative reactions?
	Tell us about a time that you decided not to go to a health care facility because you were worried that you would be treated badly for being MSM?	

Research assistants transcribed all FGD audio recordings verbatim, with translations to English (including back translations) conducted as necessary. All identifying information was removed during transcription. All transcripts were cross-verified for completeness and accuracy to correct any errors or misrepresentations that may have been made during transcription.

### Data analysis

We conducted qualitative content analysis on data generated from FGDs. In qualitative content analysis, data are categorized and reviewed iteratively to determine findings based on both the written text and the unwritten subtexts [55, 56]. Data were managed, coded and analyzed using Dedoose 8.3. Open coding was used to draft a thematic codebook. Open coding is a process in which codes are developed and applied in-real time during the process of reviewing transcripts. A team of five coders individually applied the codebook to the same four transcripts, meeting after each transcript to review the line-by-line coding, discuss discrepancies, update the codebook, and ensure consistency in coding. Remaining transcripts were coded using the final codebook. Code reports were produced by querying the qualitative database in Dedoose to retrieve all selected text associated with the codes that were related to sexual stigma. Four study authors open-coded the reports to identify text that was relevant to the questions, “How is sexual stigma manifested in this text/subtext?” and “At what level(s) is the sexual stigma occurring?” After all of the reports were coded, the study team then met to consolidate the codes that had substantive conceptual overlap, removing those that were superficial and creating clusters of codes that were closely aligned but represented distinct sexual stigma experiences. Through an iterative process of re-immersion in the data, discussion, and code refinement the team reached consensus on the key manifestations of stigma and the levels at which they were present. Our final step was the creation of a data matrix display (Table 2) to describe the distribution of sexual manifestations across type and socioecological level [56].

**Table 2:** Distribution of sexual stigma manifestation occurrences across socioecological levels

Sexual Stigma Manifestations	Socioecological Levels				
	Community	Institutions	Peers	Family	Self
Social, cultural, and religious blaming and shaming	X	X	X	X	X
Verbal abuse and intrusive questioning	X	X	X	X	X
Gossiping and outing	X	X	X	X	
Physical abuse	X		X	X	
Internal conflict and guilt about sexual behavior	X		X		X
Non-verbal judgmental gestures	X	X			
Living in constant fear and stigma avoidance	X	X			
Poor-quality services		X			

Note: Empty cells do not indicate a finding of the absence of stigma at these levels. Instead, participants did not explicitly mention sexual stigma manifestations occurring at that level.

## Results

### Range and distribution of sexual stigma manifestations

We identified a range of eight manifestations of sexual stigma. These manifestations were:(1) gossiping and outing; (2) verbal abuse and intrusive questioning; (3) non-verbal judgmental gestures; (4) societal, cultural, and religious blaming and shaming; (5) physical abuse; (6) poor-quality services; (7) living in constant fear and stigma avoidance; and (8) internal ambivalence and guilt about sexual behavior. We also found that sexual stigma manifestations were unevenly distributed across multiple socioecological levels (Table 3). Seven of the eight manifestations of sexual stigma occurred at the community level, with the exception of poor-quality services, which only occurred at the institutional level. There were no reports of physical abuse or internal conflict at the institutional level. Enacted sexual stigma was experienced across all levels, except the individual level (Table 3). *Gossiping and outing* was the most prevalent (*n* = 8) sexual stigma manifestation, experienced in the perceived, enacted, and anticipated forms and occurring across community, institutional, friends, and family levels. Additionally, sexual stigma manifested most frequently in the enacted (*n* = 6) and anticipated (*n* = 6) forms. However, four of the manifestations (i.e., *gossiping and outing, verbal abuse and intrusive questioning, non-verbal judgmental gestures, and poor-quality services*) were common between the enacted and anticipated forms.

**Table 3:** Sexual stigma manifestations arranged by form of stigma and distribution across multiple socioecological levels

Forms	Socioecological Levels				
	Community	Institution	Peers	Family	Self
Perceived	GO	GO, NV, VA, BS, PQ	--	--	--
Enacted	BS, GO, NV, PA, VA	BS, GO, NV, PQ	GO, BS, VA	BS, GO, VA, PA	--
Internalized	IC, CF	CF	IC	--	BS, IC, VA
Anticipated	CF, GO, PA, VA	CF, GO, NV, PQ, VA	CF	--	--

Note: Empty cells do not indicate a finding of the absence of stigma at these levels. Instead, participants did not explicitly mention sexual stigma manifestations occurring at that level.

Note BS = social, cultural and religious blaming and shaming. CF = living in constant fear and stigma avoidance. GO = gossiping and outing. IC = internal conflict and guilt about sexual behavior. NV = non-verbal judgmental gestures. PA = physical abuse. PQ = poor-quality services. VA = verbal abuse and intrusive questioning.

### Gossiping and outing

Gossiping was defined as spreading information, either true or false, about a person’s sexuality without their consent. MSM experienced anticipated, perceived, and enacted gossip and outing in their day-to-day lives. These experiences included gossip due to others’ perceptions of their mannerisms, for example, in the ‘feminine’ way they spoke, walked, and dressed, which was viewed in the community as a proxy-indicator that a man engaged in same-gender sexual practices. Community members also spread gossip related to participants’ social networks, including inuendo regarding the relative infrequency of the participants being observed with women compared to the time they were seen to spend in the company of other men.

Gossiping can include directly outing an individual’s sexuality in a situation and within a specific context, but it can also lead to outing in other contexts of the participants’ lives as the gossip cascades and transmits the sexual stigma into other socioecological levels. The sample quote below illustrates how gossip that originated at one socioecological level penetrated across levels through the constant spread of information. One study participant narrated his experience of being outed in a situation between his friends and family:

*“I’ve had an experience, and it happened in a store at our junction. Two girls who happens to be my friend outed me to my sister and mom when they met at the salon that I am gay. My family confronted me, but I denied to what they asked me.”* (Accra, FGD 2, participant 1).

In the example, the *gossip and outing* manifested at both the institutional level (“at the salon”) and the community level (“at our junction”). It was first enacted by two of his friends who transmitted the sexual stigma at the level of the participant’s family, where it was then re-enacted upon him through direct confrontation by the mother and sister. This process of reverberating gossip and outing can lead to rumination among MSM regarding the fear that gossip about them will continue to spread and negatively affect other aspects of their daily lives, including alienation by peers who may avoid social contact with them in order to avoid being the target of gossip and outing by association.

### Verbal abuse and intrusive questioning

Participants provided numerous examples of how sexual stigma manifested as verbal abuse. These examples include insulting, mocking, name calling, expression of disapproving thoughts, and verbal threats of harm (physical and social). Enacted verbal abuse occurred at the community, family, and friends levels. Derogatory epithets, such as ‘girl-boy’, ‘Sodom and Gomorrah’, and ‘drenches of Armageddon’ characterized the types of language imbued with references to gender oddity, sexual deviance, and religious damnation that were used to stigmatize the men’s sexualities.

In addition to outright verbal abuse, participants described being intrusively questioned about their sexuality. This manifestation occurred across community, institutional, family, friends, and individual levels. Intrusive questioning goes beyond collegial inquiries that are grounded in genuine curiosity about an individual; it is an interrogation pattern of questions designed to “expose” sexual identity or behavior with the goal of communicating to the men that their secret is not safe. This sexual stigma is still experienced even when the men provide answers to evade the questions or to conceal their sexuality. Enacted sexual stigma is manifested through the process of the intrusive questioning itself, regardless of whether or not the men confirm any details about their same-gender sexualities. This is evidenced in the previous quote, where the participant denied that he was gay in his self-defense against intrusive questioning from his family members. Another common example of this sexual stigma manifestation of *intrusive questioning* described by participants was when community and family members frequently interrogated the men on their marital status. Phrases such as, “Are you married?”, “Where is your wife?”, “Why aren’t you married?”, “But do you have a girlfriend?”, and “Don’t you want children?” seem benign on the surface (at the level of the literal text). However, the men reported that there are important cultural and religious subtexts that underlie these questions about marital interests. In the subtext of these questions is a search to uncover the real story, which is premised on the questioner’s skepticism about a man’s heterosexuality, their awareness of the socially devalued status of a suspected MSM and the corresponding awareness of the tacit social power it affords them to subject a suspected MSM to questioning and expect their cooperation.

### Non-verbal judgmental gestures

Non-verbal judgmental gestures were described as body movements that conveyed disapproval of same-gender sexuality, including acts of staring, tooth sucking, and pointing fingers. Participants reported these non-verbal gestures occurring at the community and institutional levels. The men reported that enduring long stares made them uncomfortable, and the often perceived such acts as judgmental, regardless of the starers’ intention. This discomfort was particularly acute when it occurred in the context of trying to seek services at an organization (e.g., register for educational classes, apply for a job, attend a medical appointment). The example below highlights the internal tension of being subjected to staring without a corresponding verbal narrative to corroborate the MSM’s perception that he is being judged negatively. In the following quote, the participant acknowledges that there is non-verbal nuance (subtext) present that makes it clear that he is being socially examined. In response to a prompt asking the FGD participants to elaborate on why they feel that someone looking at them is a manifestation of sexual stigma, one participant said:

*“Yes, I think it’s mostly people just seeing you and then making assumptions about who you can be and then they don’t say anything but the kind of look they give you….”* (Accra, FGD 3, participant 2).

The participants also considered staring to be intrusive and an invasion of their privacy. Those who self-identified as being ‘feminine’ anticipated receiving non-verbal judgmental gestures whenever they were seeking to obtain a service in a public venue. In the respondent’s view below, a feminine man will be subjected to sexual stigma because his femininity will be construed as evidence of his same-gender sexuality, which will be a basis for neither warmly receiving his patronage nor providing service in a manner that conveys that he is welcome.

*“It wouldn’t be friendly [because] they will find him weird, like how can a guy look feminine, dress feminine, that kind of stuff.”* (Accra, FGD4, participant 3).

This quote reinforces that participants’ experiences of sexual stigma can be compounded in situations where the sufficiency of their masculinity (or their performance of masculinity) is also scrutinized.

### Societal, cultural, and religious blaming and shaming

Participants revealed that they were blamed and shamed for unraveling traditional Ghanaian cultural values by, for example, converting other men to homosexuality. They were also blamed as being responsible for driving the epidemic spread of HIV in Ghanaian communities. Blaming and shaming occurred across community, institutional, family, and peer levels. Blaming and shaming were most frequently reported as enacted sexual stigma.

Cultural and religious alienation was a dominant mode through which this blaming and shaming was enacted and internalized by MSM. The enactment of alienation and estrangement led to the belief that due to their sexuality, they were not a legitimate member of Ghanaian society, religions, and cultures. One participant spoke about being disowned by family members and banned from using the family name because the family considered same-gender sexual activity sinful. Another participant explained that his family ridiculed him for his sexuality, claiming that MSM behaviors are against their culture:

*“They would say how can an Akan man do this, do that? At that moment, I felt so bad. Then I started to ask myself questions like, how come I got into this kind of act?”* (Accra, FGD 4, participant 4).

The risk of cultural and religious alienation also affected their sexual partners, who sometimes also internalized that their same-gender sexual desires and identities could not co-exist with their cultural and religious identities. In the quote below, another participant recounted a scene that unfolded after a sexual encounter in which the sexual partner began to experience conflict between the sexual intercourse that occurred and his experience of himself as the son of a clergy leader. The partner attempted to reconcile his conflict by blaming the participant for inducing him into forbidden sexual temptation.

*“I just remembered the experience I had with a guy. We met in a tailoring shop and then there was this shirt I needed to sew. I wanted it. So he was also interested in what I chose and he was all over me. You know every time I say something he is there and all that, so we ended up exchanging numbers, and one day he just came around and things happened. But at the end of it, he then turned around and said he was a pastor‘s son and then was blaming me for making him sin. That made me feel so bad. In fact, I felt so guilty I thought I was the worst person on Earth to have done that to him. But he did enjoy it, he never said stop until the end and then he turned round and blamed me for it. So, that made me feel really bad.”* (Accra, FGD 2, participant 5).

Religious lecturing was also utilized to alienate MSM and reinforced to them that same-gender sexual behavior was incompatible with Christianity and Islam, the two most-practiced religions in Ghana. MSM described being forced to go to religious services by family members and taken to the pastor or imam to be ‘monitored’, or ‘prayed for’. Participants also described ‘deliverance rituals’, including visits to fetish priests who are local indigenous spiritual practitioners who mediate between deceased tribal ancestors and the living world.

In addition to religion-based strategies, participants described being subjected to secular tactics. One participant described his friend’s experience with his family’s attempts to change him and the powerful effects of the shame that resulted:

*“Couple of times his dad’s call comes always after he is done with sex, and then the dad will call and would want to talk to him about [this topic] that we have been talking about. ‘Are you changing? Are you getting married?’ and it’s a bit tough for him. Like, the dad has advised him to ‘Get married so that when you are hard in the night, you won’t go and chase men, your wife is by you.’ And coincidentally the call always comes after he is done with sex or sometimes when the person is there and they are about to have sex. So it puts a lot of guilt on him. Actually, he has tried to commit suicide a couple of times simply because of that.”* (Accra, FGD 3, participant 6).

The tactics outlined in the above-mentioned quote were presented by the father as a pragmatic “solution” to the participant’s same-gender sexual attraction “dilemma,” which the father (not the participant) concluded required behavioral self-regulation. The participants experienced these tactics as controlling and pressure on them to conform to expected cultural norms, including changing their sexuality or getting married. Moreover, these efforts to ‘convert’ them and change their behaviors were identified as major sources of psychological distress and diminished social and physical well-being for the study participants and the MSM in their social networks.

### Physical abuse

Physical abuse was defined as the intention, attempt, or act of inflicting physical harm on MSM. Participants primarily described that physical abuse of MSM occurs via being struck by or pelted with objects or being slapped. Physical abuse occurred at the institutional, community, and family levels. Physical abuse can be manifested directly by an assailant or indirectly by a provocateur. For example, participants described provocations by pastors who preach inflammatory rhetoric to community members that MSM are abominations and that their persecution is sanctioned by God, inciting abuse of MSM. In the sample excerpt from an FGD, the participant describes abuse he experienced at the hands of family members of his sexual partner:

*“I once met someone on social media. We chatted for a while and later decided to visit him. When I visited, he offered me a seat outside and we started chatting. All of a sudden, I received a slap and the slapping continued from other family members. They also seized my phone and accused me of leading their son astray. They later allowed me to leave. This experience really taught me to be careful with people I meet online and also in life.”* (Kumasi, FGD 4, participant 7).

This quote illustrates how sexual stigma manifests as physical violence and the ways in which it traverses levels (and intersects with other manifestations, e.g., blaming and shaming). It also illustrates that these acts are grounded in the socially inferior status assigned to MSM and the socially sanctioned power that allows civilians to enact property seizure (“they seized my phone”), detain (“they later allowed me to leave”), and physically punish (e.g., slapping) MSM without reasonable recourse. Physical abuse and social sanctions harmed MSM both physically and emotionally. MSM described feeling ‘less’ in their community, fearing social interactions, and ultimately self-isolating in anticipation of this type of stigma (see living in constant fear and stigma avoidance).

### Poor-quality services

There were other non-physical social consequences imposed on MSM that deprived them of their rights, privileges, and societal benefits. These manifested in the diminished quality of the services that they received in commercial sectors. For example, MSM described being refused goods by merchants at marketplaces and denied due process when they register a complaint, have a grievance, or are the subject of allegations. Below is an example from the housing sector in which a participant discusses experiencing forced eviction by his landlord based on suspicion of his sexuality.

*“My landlord called me one day and told me there is going to be a renovation in my room so I have to leave the house. On the day he gave me my balance to leave, he made me aware he was actually ejecting me because I am MSM”* (Kumasi, FGD 4, participant 8). Participants expressed that, even in the health care sector, the quality of the services they received is compromised. Participants described instances in which they were refused medical attention because health providers had personal moral objections to providing care to MSM. In one example, the potentially fatal consequences of sexual stigma when it manifests as poor-quality services in a health care context is underscored. The participant in the following quote shared a story of an MSM whose death he attributes to sexual stigma in a hospital:

*“A friend who was on admission in one of the facilities died because the aunt who also works in the facility told her colleagues in the department he was admitted to that he is an MSM, and he is dying because of that… so the nurses and doctors should not attend to. The nurses and doctors also abandoned my friend and he died. So, because of that I don’t want to ever go to that facility because of how they stigmatized my friend.”* (Kumasi, FGD 4, participant 9).

The scene depicted in this quote illustrates, once again, how various manifestations of sexual stigma can be connected. In the quote, sexual stigma first manifested as *gossip and outing*, then evolved into *blaming and shaming*, before finally manifesting as *poor-quality services*. Furthermore, this quote also illustrates how sexual stigma can traverse multiple socioecological levels (e.g., originating at the family level and then being transmitted to the institutional level).

### Living in constant fear and stigma avoidance

MSM anticipated and feared stigmatization and emotional or physical harm because of their sexual identity. To avoid these dangers, MSM took precautionary measures to protect themselves. They expressed the need to self-regulate or hide their identity to conform to societal gender norms, using practices such as dressing up to appear ‘less feminine’. These practices were commonly deployed when the men needed to seek services in public spaces. One participant explained this process:

*“I will also put on baggy jeans and a (big sized) shirt to look like a man. You need to force yourself to be [a] man. We do that for just some few minutes and when we come back home, we continue being MSM.”* (Accra, FGD 4, participant 4)

In response to the constant fear of being exposed to sexual stigma, participants described a strategy whereby they seek services later in the night to avoid stigma from other patrons who may recognize them if they visited during the daytime. Participants also described practicing a strategy that involved limiting the number of male friends surrounding them to evade ‘suspicions’ in the community. Still, others described isolating themselves from heterosexual-identified people to avoid the risk of being ‘outed’.

*“My take on this is that we should choose who we call friends wisely to avoid any confrontation from our neighbors. Most MSM always want to have new friends, and I think is not the best thing to do. We should also limit the number of friends we invite into our homes to avoid suspicions.”* (Kumasi, FGD 3, participant 10).

The quote above also illustrates the way in which some of the external forms of sexual stigma can subtly become internalized, such that MSM begin to self-police their behaviors, their expressions, and their social interactions with other men who have same-gender sexual experiences.

### Internal conflict and guilt about sexual behavior

Statements from MSM demonstrated internal doubt or confusion about their sexuality and sexual behaviors. The men periodically questioned themselves, wished they were not MSM, or entertained the idea of marrying women because of societal pressure.

The internal conflict is described as being most acute in proximity to sex activity, wherein the men report that intrusive moral thoughts interrupt the encounter. An example of this is in the quote below where the participant’s sexual desires appear to collide with his belief that sex is not an appropriate activity within same-gender relations.

*“Yesterday when I was having sex with my guy, I suddenly stopped kissing him and started asking him why we were doing that. Because we are both guys. I felt so bad. I sometimes do think about it, especially when I am alone.”* (Kumasi, FGD 2, participant 11).

MSM also expressed internalized guilt, defined as feelings of remorse, regret, and self-disapproval of same-gender sexual behavior. Guilt specifically occurred when engaging in sexual acts with persons who identified as either heterosexual or religious, as MSM felt they were ‘converting’ others.

*“I feel guilty sometimes, but the moment I felt very guilty was when I slept with the school chaplain. I felt I have sinned, and that God won’t forgive me; I will go to hell. But as time went on, and he kept on requesting for it, I became okay and I felt it was okay to move on because, he made me feel special at that moment. So that was when I felt very guilty, that one. I remember I prayed that evening for forgiveness still, but the next day he came again so I felt he also knew Satan.”* (Accra, FGD 2, participant 1).

As shown in other manifestations, the sexual stigma emanating from across the socioecological spectrum can become internalized by MSM, causing them to question their identity and their roles in communities, social institutions, families, and peer networks. Chronic feelings of guilt and internal conflict have negative impacts on the well-being of MSM. One participant summarized the negative toll that these guilt feelings and internal conflict take across multiple spheres of his social reality:

*“It affected my self-esteem, it pushed me away from making male friends. I felt like if I make male friends I will get attracted to them and it might tempt me from having sexual relations with them so I dropped out from my study group, which was an all-male study group. That affected my academics, my grades were dropping and everything but fast forward somewhere in level 300 [third year at university], I think I picked myself up, and I said you cannot beat yourself forever.”* (Accra, FGD 4, participant 12).

Despite what can sometimes be years of internalizing stigma, participants also discussed the development of coping mechanisms and learned self-acceptance. Coping mechanisms included letting go of negative thoughts, embracing meditative practices, and adopting personalized theologies in which their same-gender sexuality falls within the normal range of diverse human experiences.

*“It doesn’t affect me in any way. Because I know [it] is the will of God, and if he thinks is not good, he should find a way to change it.”* (Kumasi, FGD 2, participant 13).

The quote above highlights the salience of religion in Ghanaian life, including the lives of Ghanaian MSM. The practice of reconciling one’s religious identity with one’s sexual identity is identified as a preferred goal, whereas the wholesale rejection of religion may trigger alienation from other spiritual and religious ritual aspects of community life that are still important for sociocultural identity.

## Discussion

The purpose of this qualitative study was to describe the range in manifestations of sexual stigma toward MSM in Ghana and to describe the distribution of sexual stigma manifestations across multiple socioecological levels. Consistent with previous literature, our findings show that MSM face various forms of sexual stigma across all socioecological levels of Ghanaian society, which negatively impact their behavior and health outcomes [6, 24–26, 57, 58]. In particular, MSM mentioned eight forms of stigma enacted by others: (1) gossiping and outing; (2) verbal abuse and intrusive questioning; (3) non-verbal judgmental gestures; (4) societal, cultural, and religious blaming and shaming; (5) physical abuse; (6) poor-quality services; (7) living in constant fear and stigma avoidance; and (8) internal ambivalence and guilt about sexual behavior.

Our study found that a major form of stigma toward MSM is societal, cultural, and religious blaming and shaming. Literature shows that across Africa, and particularly in Ghana, national laws, policies, and religious institutions continue to drive stigma against MSM. Ghana has an enshrined stand against non-heteronormative sexual behaviors, sanctioned by the country’s laws and religious institutions and reflected in sexual norms and expectations among Ghanaians [6, 25, 26, 57–60]. Homosexuality is broadly seen as a sin by the major religions in Ghana, Christianity and Islam, that define the perspectives of the majority of Ghanaians around appropriate ways to engage in sex and procreation [61–64].

Fundamental anti-homosexual or pro-heterosexual sentiments along with beliefs associating homosexuality with foreign culture and immorality drive stigmatization of MSM. Along with religion, other studies also found that being MSM is considered non-Ghanaian, which often contributes to experienced and internalized stigma of MSM [65–67]. Indeed, homosexuality is often falsely reflected in media and political opinions as adopted from Western countries and a non-African practice [68], while historians and anthropologists have indicated that traditional African societies and religions demonstrated more acceptance of non-heteronormative self-expression than post-colonial African societies [69]. In African nations and communities, this externalization of same-gender sexual behaviors as a foreign value facilitates the creation of a platform to advocate against homosexuality [70].

With our participants, societal, cultural, and religious views against same-sex sexual behavior manifested in labeling MSM as individuals who have diverged from Ghanaian societal norms and in shaming them for disobeying Ghanaian tradition. These forms of stigma appeared at the community level as well as in institutional settings. Within their communities, some MSM also faced harsh sanctions, such as eviction from homes, or were refused basic privileges, such as the ability to buy food from the market, because they were MSM. At the institutional level, consistent with previous literature from Ghana and other African countries, our participants recounted several instances of enacted stigma at the HCF, such as being preached at or being denied treatment [24, 26, 59, 60, 71–73]. Participants also shared instances of enacted stigma at other institutions, including places of worship. These findings provide further evidence for the importance of implementing culturally relevant interventions to address various forms of stigma experienced by MSM at the community level and in institutional settings, including health facilities [6, 57–59, 74]. Additionally, more research is needed on stigma toward MSM at other institutions, like prisons, in Ghana.

Values defined by families, community members, religious institutions, and national laws influence individuals’ self-perception and how they act. These stigma manifestations forced MSM to feel guilty about their sexual behavior and feel pressured to assimilate into normative Ghanaian traditional beliefs. Our participants often questioned their sexual behavior and shared feelings of regret for having sex with men. These occurrences align with findings from other studies on stigma toward MSM, which shows the negative consequences of family and peer stigma on the general health and mental well-being of MSM [75]. One study reported that MSM who face rejection by their community have an increased risk of substance use, depression, suicidal attempts, and sexual health conditions [75]. Additionally, previous literature shows that experiencing and anticipating stigma from the public can evoke stigma avoidance tendencies, which can harm health-seeking behaviors and reduce utilization of health services, especially HIV testing and care services [76, 77]. Our respondents spoke about fearing being outed, mistreated, or shamed by members of society, and particularly at an HCF when receiving services, and in response, adjusting their mannerisms and appearances to conceal their identity as MSM. However, other studies report that in instances where families and communities accept and support MSM, there is an observed increase in acceptance and lowered stigma as well as reduced negative health outcomes such as depression [78, 79]. More research is needed on stigma enacted by communities and families as well as research on how these groups can assume supporting roles to counteract the effects of stigma among MSM in Ghana. Our findings of chronic experience of psychological distress from exposure to various forms of physical, emotional, and structural violence indicates a high potential for development of clinical symptoms of post-traumatic stress. Interventions that also incorporate clinical and psychotherapeutic modalities that can be used in conjunction with stigma-reduction interventions are important targets for future research. Additionally, our findings may suggest the specific need for more stigma research among health facility staff, assessing their beliefs and enacted stigma towards MSM. With the findings from this research, targeted stigma-reduction trainings should be implemented at the health facility level to decrease stigmatizing beliefs and behaviors among health workers and promote safe and supportive environments for MSM to receive care.

### Limitations

While our study is one of the few to conduct FGD directly with MSM about their experiences with stigma in the community and in HCFs, our study also had some limitations. First, it is possible that included MSM may not experience the most severe stigma, as all participants were connected in some way with advocacy organizations, therefore, findings may not be generalizable to all MSM across Ghana. Furthermore, with a small FGD sample size and lacking detailed sociodemographic information about participants, it becomes more difficult to generalize these results to all Ghanaian MSM. Lastly, questions proposed in the interviews addressed sensitive issues, and it is plausible that participants were reluctant to be forthcoming in their responses. In anticipation of this concern, our team endeavored to provide MSM with a welcoming and confidential space to share their experiences.

## Conclusion

Stigma experienced at all social ecological levels has been shown to impact both mental health and sexual health of MSM. Findings from this study highlight the need to understand more of the lived stigma experiences of MSM to develop appropriate stigma-reduction interventions. Additionally, this study reflects the extent of stigma manifestations and the importance of considering wider scale stigma-reduction interventions that address institutional-level stigma beyond the HCF, but target religious and legal institutions as well. In the broader sense, although our study did not intentionally focus on family and peer enactment of stigma, MSM frequently cited these groups as perpetrators. Therefore, more community-level stigma research and interventions are needed with a focus on the role of family and peers in stigma toward MSM in Ghana.

## Data Availability

The datasets used and/or analyzed during the current study are not publicly available due to our ethical and legal requirements related to protecting participant privacy and current ethical institutional approvals but are available from the corresponding author on reasonable request pending ethical approval.
